# Influence of Microbial Treatments on Vine Growth and Must Quality: Preliminary Results

**DOI:** 10.3390/plants13223168

**Published:** 2024-11-11

**Authors:** Nicola Mercanti, Monica Macaluso, Ylenia Pieracci, Leonardo Bertonelli, Guido Flamini, Angela Zinnai

**Affiliations:** 1 Department of Agriculture, Food and Environment, University of Pisa, Via del Borghetto 80, 56124 Pisa, Italy; nicola.mercanti@phd.unipi.it (N.M.); monica.macaluso@unipi.it (M.M.); l.bertonelli2@studenti.unipi.it (L.B.); angela.zinnai@unipi.it (A.Z.); 2 Department of Pharmacy, University of Pisa, Via Bonanno 6, 56124 Pisa, Italy; guido.flamini@unipi.it; 3 Interdepartmental Research Centre “Nutraceuticals and Food for Health”, University of Pisa, Via del Borghetto 80, 56124 Pisa, Italy

**Keywords:** Sangiovese, terroir, sustainable, vineyard, biodiversity, must, wine

## Abstract

Microorganisms play a crucial role in addressing the challenges related to the increasing detrimental effects of intensive agriculture in vineyards by contributing to various aspects, from maintaining soil health and vine vitality to influencing fermentation and the overall wine features. Among microorganisms, mycorrhizal fungi are widely distributed in both natural and agricultural ecosystems, and their mutually beneficial relationship with most terrestrial plants provides valuable ecological benefits. Nowadays, the wine industry is increasingly moving toward the production of organic wines, highlighting the need for novel and healthier strategies that prioritize both the consumer well-being and the quality of the final wine product. Following our previous study in collaboration with the Bioma SA Company (Quartino, Switzerland), the investigation was continued by extending the organic practice to the cultivation. The present work, indeed, aimed to evaluate the influence of the treatment with mycorrhizal fungi on the metabolism of “Sangiovese” grapevines. In particular, the chemical parameters, including alcohol content, pH, acidity, phenolic composition, and sulfur dioxide, were assessed on the must, while the analysis of the volatile emission was conducted both on whole and pressed grapes, on must, as well as on the grape skins. To the best of our knowledge, this is the first study investigating the mycorrhizal fungi association effect on the quality of “Sangiovese” grapes and, further, its effect on the VOCs emission.

## 1. Introduction

In recent decades, human transformation of nature, particularly in perennial monocultures, has led to a significant decline in indicators of ecosystem health and biodiversity [1] as well as in human benefits deriving from ecosystem services. Ecosystem services are defined as the conditions and processes through which natural ecosystems and their species sustain and support human life [1]. The concept of ecosystem services is the main tool for assessing the value of natural capital [2]. To mitigate the adverse effects of human-induced changes on the global environment, it is crucial that each productive sector implements practices aimed at reducing its overall impact on ecosystems and biodiversity while maintaining (or even enhancing) the beneficial flow coming from nature. The wine industry is pursuing this goal. Currently, vineyards cover 7.3 million hectares globally, producing up to 260 million hectoliters of wine to meet an estimated worldwide demand of 236 million hectoliters [1]. Several wine regions are engaging in pilot projects to incorporate ecosystem services, biodiversity, and multifunctionality into vineyard management strategies [3,4,5,6]. However, despite the importance of the microbiota in providing crucial ecosystem services, its role is often neglected in most approaches [7]. Some of the initial characteristics that emerge when tasting a wine, such as aroma, acidity, and color, are linked to the region where the physical–chemical, biological, and cultural interactions occur within the vineyard: this is commonly known as *terroir* [8,9,10]. In the concept of *terroir,* the microbial interactions are also of the utmost importance. In coming decades, the agricultural sector will face significant challenges in meeting the food demands of a growing global population. However, intensive agricultural practices that rely on mineral fertilizers, agrochemicals, and water will continue to have detrimental effects on biodiversity and ecosystems [11]. Microorganisms play a crucial role in addressing these challenges by contributing to various aspects of agriculture, from maintaining soil health and vine vitality to influencing fermentation and sensory characteristics of wine. Among microorganisms, mycorrhizal fungi are widely distributed in both natural and agricultural ecosystems, and their mutually beneficial relationship with most terrestrial plants provides valuable ecological benefits. Their association with plant roots induces changes in plant physiology, improving both nutrient uptake and the efficient use of natural soil resources, and meanwhile, it increases tolerance to biotic and abiotic factors. Mycorrhizal fungi are often responsible for altering the plant secondary metabolism, which explains the increasing interest they have attracted, and therefore, they may represent important biotechnological tools for the sustainable production of safe and healthy plant-based foods [12]. Indeed, besides being involved in the enhancement of plant product quality [13], these microorganisms could also be implicated in modifying the emission of volatile organic compounds (VOCs), important molecules responsible for the communication of plants with the surrounding environment and that play a role in many ecological networks [14]. Grapevines commonly form associations with mycorrhizal fungi under typical field conditions, leading to positive effects on plant performance associated with increased growth rates and enhanced tolerance to drought [15]. Grapevine is an important perennial crop cultivated worldwide, mainly in Mediterranean regions, of which Italy is one of the five major producers [16]. Nowadays, the wine industry is increasingly moving toward the production of organic wines, highlighting the need for novel and healthier strategies that prioritize both the consumer well-being and the quality of the final wine product [17]. Following our previous study in collaboration with the Bioma SA Company (Quartino, Switzerland), it was decided to continue the investigation by extending the organic practice to the cultivation. The present work, indeed, aimed to evaluate the influence of the treatment with mycorrhizal fungi on the grape metabolism of “Sangiovese” grapevines. In particular, the chemical parameters, including alcohol content, pH, acidity, phenolic composition, and sulfur dioxide, were assessed on the grape juice, while the analysis of the volatile emission was conducted both on whole and pressed grapes, on must, as well as on the grape skins. To the best of our knowledge, this is the first study investigating the mycorrhizal fungi association effect on the quality of “Sangiovese” grapes and, further, its effect on the VOCs emission.

## 2. Results and Discussion

Microorganisms, both in the soil and on plants, play an important role, especially in promoting sustainable and regenerative agriculture. Among microorganisms, mycorrhizal fungi have attracted the scientific community attention since they are widely associated with an improvement of the soil structure as well as with a greater nutrient availability for vines. Indeed, these microbes enhance the uptake of key nutrients and reduce the need for synthetic fertilizers, allowing a shift toward organic field practices. Sustainable viticulture emphasizes the importance of microbial diversity in the vineyard, related to the enhancement of the expression of terroir. Microorganisms contribute to this by interacting with the vines and the environment, creating a distinctive microbial signature that can be reflected in the final wine product [18]. By promoting microbial biodiversity and reducing reliance on chemical inputs, the wine industry can move toward more sustainable practices. In this context, the present study aimed to determine whether the application of mycorrhiza-based products may produce favorable outcomes in the cultivation of “Sangiovese” grapevines. By comparing two vineyard plots, one treated with Ampelos and the other untreated, the research sought to determine whether the use of beneficial microorganisms could influence the key characteristics of the grapes and the resulting must.

### 2.1. Grapes Characteristics

The weight of the control and the Bioma-treated bunches as well as of the obtained juices were compared, and the results evidenced both a higher average bunch weight and a higher juice production of the control field, as reported in Table 1.

**Figure 1:**
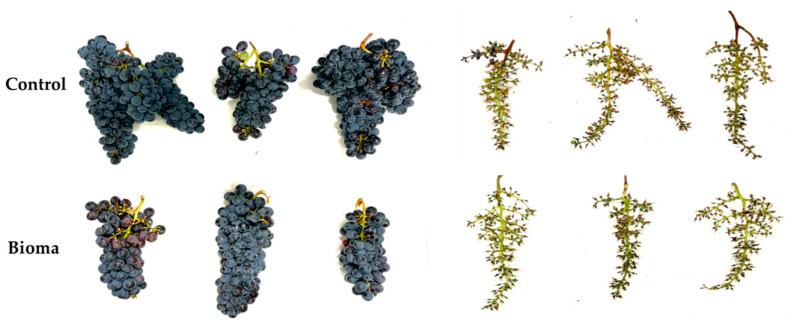
Presence of secondary ramifications in the grapes.

**Figure 2:**
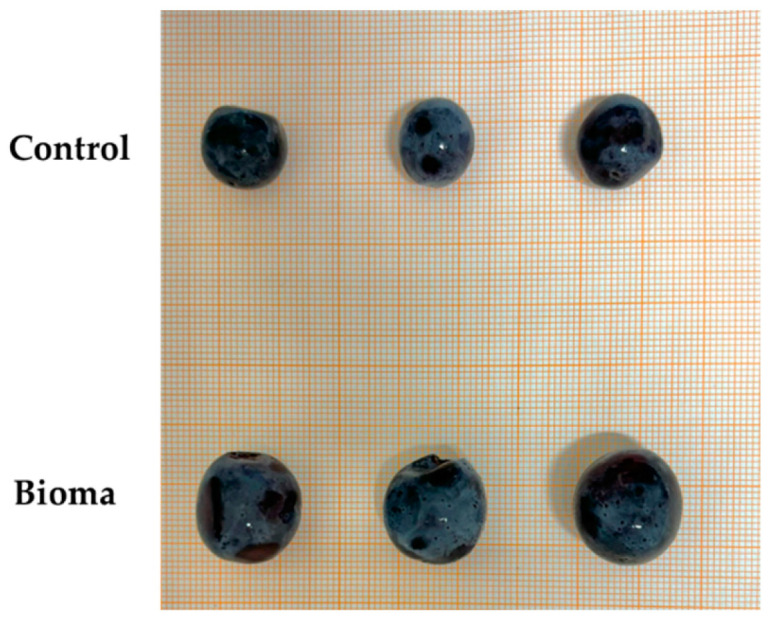
Size difference of berries.

From the visual analysis, “Bioma” grapes stems were more scattered, with a lower tendency to form secondary ramifications (Figure 1), confirming the difference in the observed weight, although the grapes were characterized by bigger berries (Figure 2). The obtained results were in contrast with Karoglan et al., who reported an improvement in bunch yield in “Cabernet Sauvignons” grapevines after treatment with mycorrhizal fungi [19]. Therefore, a lower grape yield may not be considered a negative aspect since it is often associated with increased production of secondary metabolites, crucial molecules able to strongly affect the wine quality [20]. Indeed, plants may allocate more resources to develop compounds that enhance flavor, aroma, and resistance to stress rather than focusing on the quantity of fruit produced. Moreover, the lower tendency to form secondary ramifications may represent a positive feature for ensuring a greater air recirculation inside the grape, reducing the humidity, and thus hindering the possible presence of mold while creating a more unfavorable environment for the establishment of harmful pests (*L. botrana*, *C. gnidiella* etc.) [21,22].

### 2.2. CIELAB Coordinates

For color measurement, 10 berries were randomly selected from different parts of the grape cluster and analyzed. The bloom was removed from the skin with a paper tissue. The control was significantly brighter (L* = 24.96 ± 0.005 for berries; L* = 31.25 ± 0.02 for seeds) than Bioma (L* = 14.18 ± 0.02 for berries; L* = 30.79 ± 0.06). Low L* values (lightness) generally indicate darker grapes, which typically show a higher anthocyanin content, suggesting ripeness or specific varietal characteristics [23]. In contrast, Bioma marcs showed a higher L* (22.37 ± 0.34) compared to the control marcs (13.79 ± 0.05) (Table 2). Both samples, instead, presented similar a* values, indicating a slight red tendency. The key difference between the samples was in the b* axis: The control berries shifted slightly towards blue (b* = −4.14 ± 0.02), while Bioma was almost neutral, with a minimal shift towards yellow (b* = 0.29). A similarly behavior was also evidenced for the b* value for marcs, with Bioma showing a higher b* value than the control (Table 2).

The chroma results indicated that the control sample berries had a higher chroma and a hue value closer to the blue-green region, while Bioma berries had a weaker chroma, and the hue value was closer to the yellow-red region of the color spectrum (Table 2). A hue angle close to 360° represents a transition between greenish and blueish tones. The hue angle of the control sample fell in the blue-green region of the color wheel, suggesting that the control sample may have slightly cooler tones, potentially due to grapes with more bluish or greenish shades (Table 2). In grapes, this aspect may indicate an incomplete development of the red pigmentation of certain varieties or grapes [24]. This aspect may suggest that the control sample reached a tardive adequate phenolic maturity, leading to a probable loss of fresh aroma notes. Indeed, a late ripening may affect the composition of volatile compounds. For instance, Zhao et al. observed that the ripening of “Cabernet Sauvignon” grapes significantly influences the aromatic characteristics of the wine, suggesting that a timely harvest is crucial to preserving varietal aromas [25].

**Figure 3:**
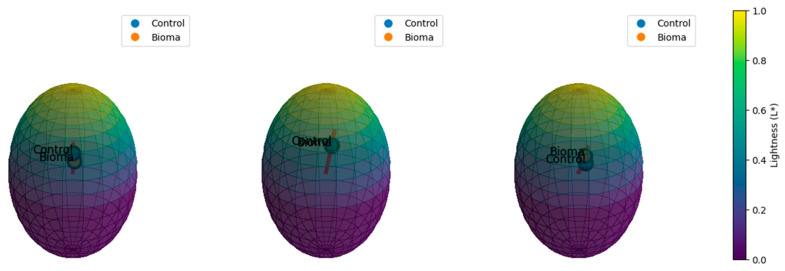
Graphical representation of Bioma and control berries in the CIELAB space in the left, graphical representation of Bioma and control seeds in the CIELAB space in the center, and graphical representation in the CIELAB space of Bioma and control marcs in the right.

Conversely, the Bioma sample had a hue angle close to 0°, placing it in the yellow-red part of the color spectrum. A hue value close to 0° is indicative of grapes with warmer tones, which could suggest a greater maturity of certain varieties or a progression towards the redder spectrum. The ΔE value is used to quantify the perceptible difference between two colors in the CIELAB color space, considering their L* (lightness), a* (green-red), and b* (blue-yellow) coordinates. The ΔE between the control and Bioma samples was 11.66, indicating a clear and significant color difference between the two samples. A ΔE value above 8 is generally considered to indicate a noticeable difference [26], meaning that the control and Bioma berries exhibited a highly perceptible color variation. Thus, the control appeared lighter with a cool blue hue, while Bioma was darker and almost neutral, with a hint of warmth towards yellow. These differences were clearly visible in the CIELAB color space, where the control berries were located closer to the blue spectrum and Bioma berries closer to the center (Figure 3). The differences in color between the Bioma and control seeds were slight, with the Bioma seed appearing barely more saturated and with a mild different shade (Figure 3). The marcs showed a clear difference between Bioma and the control, both in terms of brightness and hue, with Bioma being brighter and with more intense red tones than the control (Table 2).

### 2.3. Chemical Composition of the Grape Juice

The chemical composition of the grape juice obtained by pressing the grape berries is reported in Table 3. The results reveal significant differences between the samples treated with the mycorrhizal fungi product “Ampelos” (Bioma) and the untreated one. Notably, the Bioma-treated juice exhibited a higher concentration of organic acids, confirming the findings of Torres at al., who investigated the effect of microbial treatment on “Tempranillo” berries. Organic acids are widespread in plants, as they are involved in important metabolic pathways leading to energy production and amino acids biosynthesis and are also fundamental in the protection against osmotic stress and fruit predation. In wine, these molecules cover two relevant functions. Firstly, they are responsible for wine pH, an important parameter affecting the appearance, microbial stability, and chemical stability of the product. Secondly, they directly impact the wine taste and, in particular, the sourness [27]. Organic acids such as tartaric, malic, and citric acids derive from wine grapes, and their content seems to be affected by different factors [28]. In detail, the investigated treated juice showed a significantly elevated malic acid content (1.88 ± 0.5 g/L) compared to the control (1.19 ± 0.15 g/L) and a higher concentration of tartaric acid (3.07 ± 2.02 g/L vs. 1.92 ± 0.21 g/L). These findings suggest that mycorrhizal treatment may contribute to a more pronounced acidity profile, potentially enhancing the freshness and the structure of the wine that is produced. Conversely, no significant differences were observed in total phenol content, although the Bioma samples showed a slight reduction (2.56 ± 0.12 g/L) compared to the control (2.61 g/L) (Table 3).

Regarding anthocyanins, Bioma-treated grape juice displayed lower levels of both total (167.51 ± 4.13 vs. 203.39 ± 2.86) and bleachable anthocyanins (122.54 ± 0.30 vs. 167.51 ± 7.42 in the control), indicating that the treatment may slightly affect the wine color intensity. However, the ratio of total to bleachable anthocyanins was higher for Bioma, highlighting that the biosynthesis of the non-oxidizable content of these secondary metabolites appears to be influenced by treatment with microorganisms (Table 3). Notably, the Bioma sample exhibited higher sugar levels (183.46 ± 2.11 for Bioma and 157.15 ± 7.1 for control) suggesting a potential for wines with higher alcohol content (Table 3). Finally, treatment with Ampelos appears to have a positive effect on volatile acidity since the treated samples had no detectable acetic acid, whereas the control had a concentration of 30 ± 2.82 g/L. Indeed, the presence of acetic acid in the grapes could be associated with the grape cracking and the consequence of fermentation by indigenous microorganisms of the berry juice coming out. A high density of berries per grape could be related to a greater tendency to crushing and thus to a higher production of acetic acid, considered an undesirable compound.

### 2.4. Aroma Composition

Wine aroma, conferred by the released volatile organic compounds (VOCs), represents an important feature of wine products, as it is able to influence the consumer acceptability [29]. The volatile bouquet, besides being influenced by the vinification and the ageing processes, derives from the grape berries [2,26]. Grape variety, indeed, together with *terroir* plays a major role in determining the so called primary (or “varietal”) aroma of the wine [29]. In addition to being important for the quality of the product, VOCs are responsible for the communication of the plants with the surrounding environment [30,31], playing a role in many ecological networks [14]. This explains why the biosynthesis and the accumulation of those chemicals are not homogeneous, but rather, they are strongly affected by different factors capable of altering the expression of genes involved in different metabolic pathways [32,33,34]. In this context, since mycorrhizal fungi are increasingly used as eco-friendly techniques to protect crops from pests by activating plant mechanisms of resistance, in the present study, we investigated their effect on the volatile emission of “Sangiovese” grapes and, in particular, of whole berries, pressed berries, grape juice, and grape skins.

#### 2.4.1. Whole Grapes

The complete chemical compositions of the headspaces of the whole grapes harvested from both control and treated vines are reported in Table 4. Overall, 19 compounds were identified, mainly belonging to the class of non-terpene derivatives that were higher in the Bioma grapes compared to the control ones. In detail, within this class, 2-octanone was found as the major compound in both the samples, even though it was significantly higher in control grapes (57.0%) than in Bioma ones (28.8%). Besides this molecule, not-negligible amounts of other ketones, including 2-hexanone, 2-heptanone, 6-methyl-5-hepten-2-one, 2-nonanone, and 2-undecanone, were detected, and among these, 2-heptanone was significantly higher in the control grapes, and 2-undecanone prevailed in Bioma sample, while the other showed no significant differences. Ketones were reported by Gao et al. [35] as one of the major chemical classes detected in grape berries, with the continuous presence of 2-octanone in all the investigated varieties. Although no literature studies have suggested the influence of vineyard treatment with these components, the present study emphasized a strong effect of Bioma on the vine metabolic pathways leading to the biosynthesis of these molecules.

While control grape berries were characterized by a prevalence of ketones, those of Bioma showed greater relative amounts of the aliphatic hydrocarbon heptane and f the non-terpene alcohols, particularly represented by 1-hexanol, responsible for fruity, flowery, grassy, and green characters [36].

Yue et al. indicated this molecule as one of the most abundant alcohols present in mature grape berries, with few differences among different varieties and different vintages. Therefore, the differences highlighted in the present work could probably be linked to an enhanced metabolic activity related to the mycorrhizal fungi treatment [37].

In addition, Bioma grapes showed consistently higher relative amounts of monoterpene hydrocarbons, the only two components of which were limonene and pseudocumene. Monoterpenes have been widely reported as markers of wine varieties [38]. However, no study has yet investigated the effect of vine treatment on their metabolism. In the present study, the mycorrhizal treatment seemed to be responsible for the increased biosynthesis of those flavor compounds usually associated with a possible improvement of the aroma profile [39].

#### 2.4.2. Pressed Grapes

The complete volatile emission of the pressed grapes is reported in Table 5. Unlike whole grape berries, these samples were characterized by the presence of lower percentages of ketones, represented only by 2-octanone and 2-nonanone, which were also in this case in higher amounts in control grapes. The most abundant chemical class of the pressed grapes volatile emission was represented by aldehydes, among which the most abundant detected compound was (*E*)-2-hexenal, followed by hexanal. The treatment with mycorrhizal fungi seemed to be able to enhance the biosynthesis of the former compound to the disadvantage of the latter. Besides being associated with more pleasant fruity aroma notes [40] compared to hexanal and exhibiting fresh, green aromas [41]), (*E*)-2-hexenal is also reported to be an important precursor of varietal thiol aroma compounds. Indeed, it is able to react with glutathione, forming the aroma precursor 3-S-glutathionylhexanol [42]. This aspect suggests a probable improvement of the aroma profile of wines obtained from grapes treated with Bioma products. Along with aldehydes, alcohols were also found in relevant amounts, with (*E*)-2-hexen-1-ol and 1-hexanol as key compounds. Analogously to the aldehydes, Bioma treatment seemed to be related to increased production of more (*E*)-2-hexen-1-ol than 1-hexanol and also linked to the production of the unsaturated C6-thiol precursors of key aroma compounds.

#### 2.4.3. Grape Juice

Table 6 shows the complete volatile composition of the headspaces of the investigated grape juices. Analogously to the pressed grape berries, juice aroma was mainly characterized by aldehydes, with particular reference to hexanal and (*E*)-2-hexenal. For the grape juice, however, the differences in the relative content of those molecules between the control and the Bioma samples were milder. Indeed, although (*E*)-2-hexenal in this case was also higher in the Bioma than in the control sample, the discrepancy in their relative content was of 3%. Conversely, no significant differences were revealed for hexanal. Besides aldehydes, non-negligible amounts of alcohols were found and only represented by (*E*)-2-hexen-1-ol and 1-hexanol, both significantly lower in Bioma-treated grape juice.

The overall volatile profile of the grape juice evidences slight modification of the plant volatile metabolism, with a mild shift towards the biosynthesis of important aroma precursors that could influence the flavor and aroma during fermentation, with probable differences in the final wine product.

#### 2.4.4. Grape Skins

The volatile emission of the skins obtained from both control and Bioma-treated grapes is reported in Table 7. Grape skins showed a volatile emission mainly represented by the alcohol 1-hexanol that did not show significant differences between the samples, accounting for 86.3 and 88.2% in control and Bioma, respectively. Conversely to the pressed grapes and the grape juice, the aldehyde (*E*)-2-hexenal was higher in control grape skins, suggesting probable differential metabolic pathways in the different fruit parts as a consequence of the Bioma treatment. The presence of limonene in Bioma-treated grapes skins, even in very limited percentages, may indicate the beneficial effects of the mycorrhizal treatment, also suggesting positive effects on the aroma profile of wines obtained with sink maceration [43].

## 3. Materials and Methods

### 3.1. Field Position

The experimentation was carried out at La Cura wine-growing estate (Grosseto, Italy), where two Sangiovese fields exposed to the same climatic and topographical conditions (42°58′22.4″ N 10°48′56.9″ E for the untreated field (control) and 42°56′36.1″ N 10°49′09.1″ E for the treated field (Bioma)) were selected to minimize differences related to the environment.

### 3.2. Product Application

The Ampelos product (Bioma SA Company) was constituted as follows: 5% by weight of mycorrhizae fungi, 1 × 10^10^ CFU/g of rhizosphere bacteria (*B. subtilis*, *P. fluorescens*, and *Glomus* sp.), and 1 × 10^9^ CFU/g of Trichoderma. The product was applied on the soil as a solution of 3.25 kg/ha during the stages B, C, D, and E of the Baggiolini sheet [44]. No treatment was carried out in the control field.

### 3.3. Sampling

Visual evaluation of grapes and stalks was accomplished to verify the presence of differences in the amount of berries per grape. For the chemical analyses, six grapes were randomly sampled from both Bioma-treated and control fields. The collected berries were weighed and pressed to obtain the grape juice, which was then centrifuged (10,000 rpm for 5 min at 4 °C) and filtered. The analysis of the volatile emission was performed on whole grape berries, pressed grape berries, grape juice, and grape skin of three random grapes of each field.

### 3.4. Chemical Analyses

The chemical determination of the sugars content (hexoses g/L) as well as of the organic acids (tartaric acid (g/L), L-malic acid (g/L), L-lactic acid (g/L), citric acid (g/L), and acetic acid) was performed using an iCubio iMagic M9 analyzer (Shenzhen iCubio Biomedical Technology Co., Ltd., Shenzhen, China) operating in complete automation, as reported by Mercanti et al., 2024 [17]. Titratable acidity (tartaric acid g/L), pH, and net volatile acidity (g/L acetic acid) were carried out according to the OIV methods [45], while total phenols were measured using the Folin–Ciocalteu colorimetric assay, modified as follows: 1 mL of sample (previously diluted 1:10 with deionized water), 5 mL of Folin−Ciocalteu reagent, 15 mL of 20% sodium carbonate, and 79 mL of deionized water were mixed in a 100 mL glass flask, and after 30 min of incubation at room temperature, the absorbance of the samples was measured at 720 nm against blank. Total phenols content was expressed as g/L of catechin. Finally, total anthocyanins (g/L malvin) and bleachable anthocyanins (g/L malvin) were evaluated following the hydrochloric acid method and the bisulfite bleaching method reported by Mercanti et al. [46].

### 3.5. Determination of Wine Colour Coordinates

The evaluation of the chromatic characteristics of the grape juice was accomplished using a Benchtop CLM-196 colorimeter (Eoptis-38121 Trento (TN), Italy). The color values are described using the native CIE coordinates L*, a*, and b*, according to the official OIV-method International Organization of Vine and Wine (OIV [47]). In particular, the three parameters are described as follows:

- L*, representing brightness (0 = black, 100 = white);
- a*, which indicates the color range from green (negative values) to red (positive values);
- b*, indicating the color range from blue (negative values) to yellow (positive values).

In addition, chroma value (C) was calculated as C=$\;\sqrt{a^{*2}+b^{*2}}$ and hue (h) value as h= arctang$\left(\frac{b*}{a*}\right)$, while ΔE was evaluated as ΔE=$\sqrt{{\Delta L}^{*2}+{\Delta a}^{*2}+{\Delta b}^{*2}}$.

### 3.6. Headspace Solid-Phase Microextraction and GC-MS Analyses

The volatile emissions of the wine samples were analyzed in triplicate by using Headspace–Solid-Phase Microextraction (HS-SPME). For the analysis, 50 g of whole grapes, pressed grapes, and grape sinks were separately inserted in a 100 mL glass beaker and 25 mL of grape juice in a 50 mL glass flask. The containers were covered with aluminum foil and left to equilibrate for 30 min at room temperature. Then, the headspaces were sampled using a Supelco SPME device equipped with a divinylbenzene/carboxen/polydimethylsiloxane (DVB/CAR/PDMS) fiber (50/30/30 µm, Supelco analytical, Bellefonte, PA, USA), which was preconditioned following the manufacturer’s instructions. The sampling times were experimentally determined to obtain an optimal adsorption of the volatiles and to avoid both under- and over-saturation of the fiber and of the mass spectrometer. In detail, the whole-grapes headspace was sampled for 30 min, pressed grapes and grape skins headspaces for 20 min, and grape juice for 15 min. Once the sampling time was finished, the fiber was injected into the gas chromatography–mass spectrometry analyses apparatus (Agilent Technologies Inc., Santa Clara, CA, USA) equipped with an Agilent HP-5MS capillary column (30 m × 0.25 mm; coating thickness 0.25 µm) and an Agilent 5977B single quadrupole mass detector.

### 3.7. GC-MS Analyses

The Gas Chromatography–Electron Ionization Mass Spectrometry (GC-EIMS) analyses were carried out with an Agilent 7890B gas chromatograph (Agilent Technologies Inc., Santa Clara, CA, USA) equipped with an Agilent HP-5MS capillary column (30 m × 0.25 mm; coating thickness 0.25 µm) and an Agilent 5977B single quadrupole mass detector. The injection was performed with the splitless method using an injector temperature of 250 °C. The analytical conditions were set as follows: oven temperature rising from 60 to 240 °C at 3 °C/min; transfer line temperature at 240 °C; carrier gas helium at 1 mL/min. The acquisition parameters were the following: full scan; scan range: 30–300 *m*/*z*; scan time: 1.0 s. The peak identification relied on a comparison between the retention times with those of the authentic samples, comparing their linear retention indices relative to the series of *n*-hydrocarbons (C8–C27) and a computer matching against commercial (NIST 14 and ADAMS 2007) and laboratory-developed mass spectra libraries built up from pure substances and components of commercial essential oils of known composition and the MS literature data [48,49,50,51,52,53].

### 3.8. Statistical Analyses

Significant differences in the compositional parameters as well as in the volatile emission of the investigated samples were assessed by *t*-test using a *p* ≤ 0.05. The statistical treatment was performed with JMP Pro 14.0 statistical package (SAS Institute; Cary, NC, USA).

## 4. Conclusions

The preliminary results of this study showed that field treatment with Ampelos’ product had a positive impact on grape ripening and must quality in “Sangiovese” vineyards. The treatment led to an increase in organic acidity, particularly malic and tartaric acids, and a higher concentration of sugars, suggesting a potential increase in the alcohol content of the deriving wine. From an aromatic point of view, the treatment improved the complexity of the profile, with a greater presence of fruity and citrus notes that could enrich the sensory characteristics of the future wine. These effects, combined with a possible reduction in humidity and disease risk due to an improvement of the air circulation within the treated grape berries, suggests that the use of microorganisms could significantly contribute to a more sustainable vineyard management by maintaining soil health and protecting vines from diseases while preserving the sensory qualities of the wine. Nevertheless, future research is necessary to monitor the fermentation and refinement processes in wine obtained from Bioma-treated grapes as well as to address the microbial pattern of the soil.

## Figures and Tables

**Table 1:** Bioma and Control grapes characteristics.

Samples	N° Grapes	Average Grape Weight (g)		Total Grapes Weight (g)		Must Weight (g)
Bioma	6	242.50 ± 117.06 ^B^		1455.05		1959
Control	6	426.02 ± 117.21 ^A^		2556.16		2174
**Samples**	**Average Grape Berries (N°)**		**Total Berries Weight (g)**		**Average Berry Weight (g)**	
Bioma	104.5 ± 33.39 ^B^		1379.85		2.20	
Control	230.6 ± 67.63 ^A^		2394.22		1.72	

Superscript uppercase letters (A–B) indicate statistically significant differences in the relative abundances of the chemical compounds among the samples, *p* < 0.05.

**Table 2:** CIELAB coordinates of Bioma and control samples.

Samples	L*	a*	b*	Chroma	Hue (°)	ΔE_ab_
**Bioma berries**	14.18 ± 0.02 ^B^	2.81 ± 0.02 ^B^	0.29 ± 0 ^A^	2.83 ± 0.02 ^B^	5.90 ± 0.04 ^B^	≈11.66
**Control berries**	24.96 ± 0.005 ^A^	3.12 ± 0 ^A^	−4.14 ± 0.02 ^B^	5.18 ± 0.015 ^A^	306.79 ± 0.14 ^A^	
**Bioma seeds**	30.79 ± 0.06 ^B^	6.14 ± 0.011 ^A^	12.35 ± 0.035	13.79 ± 0.03	63.56 ± 0.03 ^B^	≈0.94
**Control seeds**	31.25 ± 0.02 ^A^	5.32 ± 0.01 ^B^	12.43 ± 0.29	13.52 ± 0.26	66.79 ± 0.51 ^A^	
**Bioma marcs**	22.37 ± 0.34 ^A^	13.06 ± 0.20 ^B^	0.86 ± 0.086 ^A^	15.47 ± 0.20 ^B^	3.75 ± 0.31 ^A^	≈8.93
**Control marcs**	13.79 ± 0.05 ^B^	15.46 ± 0.07 ^A^	0.17 ± 0.60 ^B^	13.78 ± 0.07 ^A^	0.64 ± 0.22 ^B^	

Superscript uppercase letters (A–B) indicate statistically significant differences in the relative abundances of the chemical compounds among the samples; *p* < 0.05.

**Table 3:** Chemical composition of grape juice of treated field (Bioma) and untreated field (control).

Samples	Lactic Acid (g/L)	Citric Acid (g/L)	Malic Acid (g/L)	Tartaric Acid (g/L)	Acetic Acid (g/L)	pH	Titratable Acidity (g/L of Tartaric Acid)	Volatile Acidity (g/L of Acetic Acid)
**Bioma**	0.1165 ± 0.017	0.07 ± 0.025	1.88 ± 0.5 ^A^	3.07 ± 2.02	- ^B^	3.71 ± 0.14	4.53 ± 0.55	0.12 ± 0
**Control**	0.113 ± 0	0.0335 ± 0	1.19 ± 0.15 ^B^	1.92 ± 0.21	30 ± 2.82 ^A^	3.65 ± 0.02	3.87 ± 0.28	0.11 ± 0.03
**Samples**	**Total Phenols (g/L of Catechins)**	**Total Anthocyanins (TA) (mg/L of Malvin)**	**Bleachable Anthocyanins (BA) (mg/L of Malvin)**	**TA/BA**	**Hexoses (g/L)**	**Glucose (g/L)**	**Fructose (g/L)**	**Glucose/** **Fructose**
**Bioma**	2.56 ± 0.12	167.51 ± 4.13 ^B^	122.54 ± 0.30	1.36 ^A^	183.46 ± 2.11 ^A^	92.04 ± 1.26	91.41 ± 0.85 ^A^	1.006
**Control**	2.61 ± 0	203.39 ± 2.86 ^A^	167.51 ± 7.42	1.21 ^B^	157.15 ± 7.19 ^B^	77.35 ± 5.25	79.80 ± 1.90 ^B^	0.96

Superscript uppercase letters (A–B) indicate statistically significant differences in the relative abundances of the chemical compounds among the samples; *p* < 0.05.

**Table 4:** Complete volatile profile of the whole grape berries.

Compounds	l.r.i. ^1^	Class	Relative Abundance (%) ± Standard Deviation		Significance Level
			Control Grapes	Bioma Grapes	
Heptane	700	nt	7.2 ± 0.53 ^B^	25.6 ± 3.44 ^A^	*
2-Hexanone	790	nt	2.1 ± 0.02	2.0 ± 0.09	n.s.
(*Z*)-3-Hexen-1-ol	896	nt	0.4 ± 0.11	1.8 ± 0.11	***
1-Hexanol	903	nt	2.7 ± 0.28 ^B^	10.7 ± 0.17 ^A^	***
2-Heptanone	926	nt	13.7 ± 1.18 ^A^	5.0 ± 0.06 ^B^	**
1-Octen-3-ol	976	nt	1.1 ± 0.13 ^B^	2.1 ± 0.00 ^A^	**
6-Methyl-5-hepten-2-one	986	nt	1.5 ± 0.18	2.5 ± 1.07	n.s.
2-Octanone	990	nt	57.0 ± 0.11 ^A^	28.8 ± 4.85 ^B^	**
Pseudocumene	990	nt	1.1 ± 0.11 ^B^	1.8 ± 0.19 ^A^	*
3-Ethyl-1-hexanol	1030	nt	- ^2,B^	1.2 ± 0.17 ^A^	**
Limonene	1029	mh	- ^B^	3.5 ± 0.19 ^A^	**
1-Octanol	1069	nt	0.6 ± 0.04 ^A^	- ^B^	*
2-Nonanone	1093	nt	5.2 ± 0.97	5.2 ± 0.77	n.s.
Nonanal	1105	nt	2.2 ± 0.01 ^A^	1.1 ± 0.22 ^B^	*
Methylundecane	1156	nt	0.7 ± 0.08 ^B^	1.4 ± 0.07 ^A^	*
Dodecane	1200	nt	1.1 ± 0.12 ^B^	3.7 ± 0.26 ^A^	**
Decanal	1206	nt	2.2 ± 0.08	1.9 ± 0.65	n.s.
2-Undecanone	1294	nt	0.5 ± 0.2 ^B^	1.6 ± 0.19 ^A^	**
(*E*)-Geranylacetone	1453	ac	0.6 ± 0.13 ^A^	0.3 ± 0.00 ^B^	**
Other non-terpene derivatives (nt)			98.2 ± 0.25	94.6 ± 0.01	*
Monoterpene hydrocarbons (mh)			1.1 ± 0.11	5.3 ± 0	***
Apocarotenoids (ac)			0.6 ± 0.13	0.3 ± 0	*
Total identified (%)			100 ± 0.01	100 ± 0.01	

^1^ Linear retention index on a HP 5-MS capillary column; ^2^ not detected. Superscript uppercase letters (A–B) indicate statistically significant differences between Bioma and control samples. The asterisks in the “Significant level” column indicates * *p* < 0.05, ** *p* < 0.01, *** *p* < 0.0001, while n.s. means not significant.

**Table 5:** Complete volatile profile of the pressed grape berries.

Compounds	l.r.i. ^1^	Class	Relative Abundance (%) ± Standard Deviation		Significance Level
			Control Pressed Grapes	Bioma Pressed Grapes	
Hexanal	800	nt	23.8 ± 0.53 ^A^	15.9 ± 0.98 ^b^	**
(*E*)-2-Hexenal	854	nt	27.5 ± 0.07 ^B^	40.5 ± 0.72 ^A^	**
(*E*)-2-Hexen-1-ol	862	nt	19.4 ± 0.09 ^B^	27.4 ± 0.73 ^A^	**
1-Hexanol	871	nt	26.3 ± 0.75 ^A^	14.8 ± 1.07 ^B^	**
2-Octanone	990	nt	1.1 ± 0.02 ^A^	0.2 ± 0.02 ^B^	***
(*E*)-2-hexenyl acetate	1017	nt	0.4 ± 0.01 ^B^	0.7 ± 0.04 ^A^	**
1-Octanol	1069	nt	0.1 ± 0.00	- ^2^	n.s.
2-Nonanone	1093	nt	0.2 ± 0.00 ^A^	- ^B^	**
Nonanal	1105	nt	0.6 ± 0.02 ^A^	0.3 ± 0.02 ^B^	***
Decanal	1206	nt	0.5 ± 0.01 ^A^	0.2 ± 0.02 ^B^	***
Other non-terpene derivatives (nt)			100 ± 0.01	100 ± 0.00	n.s.
Total identified (%)			100 ± 0.01	100 ± 0.00	

^1^ Linear retention index on a HP 5-MS capillary column; ^2^ not detected. Superscript uppercase letters (A–B) indicate statistically significant differences between Bioma and control samples. The asterisks in the “Significant level” column indicates ** *p* < 0.01, *** *p* < 0.0001, while n.s. means not significant.

**Table 6:** Complete volatile profile of the grape juices.

Compounds	l.r.i. ^1^	Class	Relative Abundance (%) ± Standard Deviation		Significance Level
			Control Grape Juice	Bioma Grape Juice	
Acetic acid	610	nt	0.2 ± 0.05	0.2 ± 0.04	n.s.
Heptane	700	nt	0.2 ± 0.02 ^A^	0.1 ± 0.02 ^B^	**
Hexanal	800	nt	30.7 ± 0.22	31.2 ± 0.24	n.s.
Hex-2-enal	851	nt	0.8 ± 0.07	0.9 ± 0.06	n.s.
(*E*)-2-Hexenal	854	nt	56.2 ± 0.14 ^B^	59.2 ± 0.16 ^A^	***
(*E*)-2-Hexen-1-ol	862	nt	6.2 ± 0.36 ^A^	3.3 ± 0.21 ^B^	**
1-Hexanol	871	nt	5.5 ± 0.09 ^A^	4.7 ± 0.2 ^B^	*
Nonanal	1104	nt	0.1 ± 0.01 ^B^	0.3 ± 0.03 ^A^	**
Other non-terpene derivatives (nt)			100 ± 0.01	100 ± 0.00	n.s.
Total identified (%)			100 ± 0.01	100 ± 0.00	

^1^ Linear retention index on a HP 5-MS capillary column. Superscript uppercase letters (A–B) indicate statistically significant differences between Bioma and control samples. The asterisks in the “Significant level” column indicates * *p* < 0.05, ** *p* < 0.01, *** *p* < 0.0001, while n.s. means not significant.

**Table 7:** Complete volatile profile of the grape skins.

Compounds	l.r.i. ^1^	Class	Relative Abundance (%) ± Standard Deviation		Significance Level
			Control Grape Sink	Bioma Grape Sink	
2-Pentanone	685	nt	0.8 ± 0.11 ^B^	1.5 ± 0.06 ^A^	**
Hexanal	800	nt	3.6 ± 1.33	1.0 ± 0.38	n.s.
(*E*)-2-Hexenal	854	nt	7.2 ± 1.43 ^A^	4.1 ± 0.61 ^B^	*
1-Hexanol	871	nt	86.3 ± 2.85	88.2 ± 0.77	n.s.
1-Octen-3-ol	981	nt	0.2 ± 0.00 ^B^	0.3 ± 0.02 ^A^	**
6-Methyl-5-hepten-2-one	986	nt	0.4 ± 0.07 ^A^	- ^2,B^	*
2-Octanone	990	nt	0.2 ± 0.03 ^B^	0.8 ± 0.09 ^A^	**
2-Pentylfuran	993	nt	0.8 ± 0.11	0.7 ± 0.02	n.s.
Hexyl acetate	1011	nt	-	0.6 ± 0.33	n.s.
(*E*)-2-hexenyl acetate	1016	nt	0.2 ± 0.07	2.2 ± 1.36	n.s.
Limonene	1029	mh	- ^B^	0.2 ± 0.04 ^A^	*
Nonanal	1105	nt	- ^B^	0.1 ± 0.01 ^A^	**
Phenethyl alcohol	1116	nt	0.1 ± 0.01	0.1 ± 0.01	n.s.
Decanal	1205	nt	0.4 ± 0.06 ^A^	0.2 ± 0.00 ^B^	*
Other non-terpene derivatives (nt)			100.0 ± 0.00 ^A^	99.8 ± 0.04 ^B^	**
Monoterpene hydrocarbons (mh)			- ^B^	0.2 ± 0.04 ^A^	*
Total identified (%)			100 ± 0.00	100 ± 0.00	

^1^ Linear retention index on a HP 5-MS capillary column; ^2^ not detected. Superscript uppercase letters (A–B) indicate statistically significant differences between Bioma and control samples. The asterisks in the “Significant level” column indicates * *p* < 0.05, ** *p* < 0.01, while n.s. means not significant.

## Data Availability

The original contributions presented in the study are included in the article; further inquiries can be directed to the corresponding author.
